# mRNA expression data in breast cancers before and after consumption of walnut by women

**DOI:** 10.1016/j.dib.2019.104050

**Published:** 2019-05-23

**Authors:** W. Elaine Hardman, Donald A. Primerano, Mary T. Legenza, James Morgan, Jun Fan, James Denvir

**Affiliations:** a Joan C. Edwards School of Medicine, Marshall University, Department of Biomedical Sciences, 1600 Medical Center Dr., Huntington, WV, 25701, USA; b Edwards Comprehensive Cancer Center, 1400 Hal Greer Drive, Huntington, WV, 25701, USA; c St. Mary's Cancer Center, 2900 1st Avenue, Huntington, WV, 25702, USA

**Keywords:** Breast cancer, Walnut consumption, mRNA expression

## Abstract

This article contains supporting data for the research paper entitled: *‘*Dietary walnut altered gene expressions related to tumor growth, survival, and metastasis in breast cancer patients: a pilot clinical trial’ [1] Hardman et al., 2019. Included are tables for all mapped genes and all unmapped loci identifications that were significantly changed in breast cancers by consumption of walnut for about 2 weeks. All gene networks that were identified by Ingenuity Pathway Analyses as modified are shown in table 3. Files containing the raw reads, along with a shell script describing the complete data analysis pipeline, were deposited to the Gene Expression Omnibus (GEO) at the National Center for Biotechnology Information (NCBI) and can be obtained via accession number GSE111073. https://www.ncbi.nlm.nih.gov/geo/query/acc.cgi?acc=GSE111073.

**Table:** Specifications table

Subject area	*Breast cancer*
More specific subject area	*Diet supplementation with walnut*
Type of data	*Tables of modified genes and gene pathways*
How data was acquired	*Next-gen sequencing of mRNA from breast cancer tumors* Total RNA was extracted from biopsy or surgical specimens using RNeasy, Lipid Tissue kit (Qiagen.com). RNA sample quality was assessed on RNA Pico chips in using an Agilent 2100 Bioanalyzer (Agilent, Santa Clara, CA). One microgram of total RNA was used to construct RNA-Seq libraries using a TruSeq stranded total RNA library prep kit with RiboZero(H∖M∖R) ribosomal RNA reduction (Illumina Inc., San Diego, CA) according to the kit's instructions. Twenty RNA-Seq libraries were clustered on an Illumina cBot and sequenced on a HiSeq 1500 platform, in a 2 x 50 base paired end design yielding a minimum of 50 million reads per sample. Reads were trimmed using Trimmomatic v 0.36 [2] to remove low-quality base calls and adapter sequences, and then aligned to the human reference genome GRCh38 using HISAT v2.1.0 [3]. Resulting bam files were sorted and indexed with SamTools v1.3.1 [3], [4], and PCR and optical duplicate reads marked using Picard tools v2.6.0. The numbers of reads mapping to each gene for each sample were counted using the R/Bioconductor package GenomicAlignments, v1.12.2 [5] and the Ensembl gene database for GRCh38, build 84 [6]. Differential gene expression was computed using DESeq2 version 1.10.0 [7], with a statistical model comparing the ratio of expression between surgery and biopsy specimens for the walnut-consuming group to the ratio of expression between surgery and biopsy specimens for the control group.
Data format	Raw expression reads were analyzed by Ingenuity Pathway to identify changed gene expressions in the tumor by walnut consumption.
Experimental factors	Women with breast cancer in the walnut group consumed 2 ounces of walnuts per day for 2–3 weeks, the control group did not consume walnut.
Experimental features	The data were obtained in a non-placebo, two-arm, clinical trial. Women with breast lumps large enough for research and pathology biopsies were recruited and randomized to walnut consuming or control groups. Immediately after biopsy collection, women in the walnut group began to consume two ounces of walnuts per day until follow-up surgery, the control group did not consume walnut. Pathology confirmed that lumps were breast cancer in all women who remained in the trial. At surgery, about two weeks after biopsy, additional specimens were taken from the breast cancers. Changes in gene expression in the surgical specimen compared to baseline biopsy were determined in each individual woman in walnut-consuming (n = 5) and control (n = 5) groups. RNA-Seq was performed. Resulting expression data were analyzed by Ingenuity pathway analyses.
Data source location	Huntington, West Virginia
Data accessibility	Files containing the raw reads, along with a shell script describing the complete data analysis pipeline, were deposited to the Gene Expression Omnibus (GEO) at the National Center for Biotechnology Information (NCBI) and can be obtained via accession number GSE111073. https://www.ncbi.nlm.nih.gov/geo/query/acc.cgi?acc=GSE111073
Related research article	Hardman WE, Primerano DA, Legenza MA, Morgan J, Fan J, Denvir J. Dietary walnut altered gene expressions related to tumor growth, survival, and metastasis in breast cancer patients: a pilot clinical trial. Nutr Research 2019; In Press. https://doi.org/10.1016/j.nutres.2019.03.004

**Table:** 

**Value of the data This data can be of value to those who:** • desire to further investigate synergism between diet and cancer therapy • desire to understand why some cancers responded to the dietary intervention and some may not • desire to discover beneficial combinations of dietary components and/or standard cancer therapies by understanding the genes that are influenced

## 1 Data

The data in Table 1 present the known genes in which the log ratio of [(gene expressions in the breast tumor at surgery) divided by (the expression of that gene in the initial biopsy)] in the subjects who consumed walnut divided by the [(gene expressions in the breast tumor at surgery) divided by (the expression of that gene in the initial biopsy)] of that gene in the control subjects were significant. Thus, this data shows the genes in which mRNA expression was significantly changed by consumption of walnut compared to control and the direction of that change (increased or decreased).

**Table 1:** All mapped loci. ^©^ 2000–2018 QIAGEN. All rights reserved.

Expr log ratio	Expr false discovery rate (q-value)	ID	Flags	Symbol	Entrez gene name	Location	Type(s)	Drug(s)
28.23	5.54E-03	ENSG00000255277		ABCC6P2	ATP binding cassette subfamily C member 6 pseudogene 2	Other	other	
27.99	5.54E-03	ENSG00000144827		ABHD10	abhydrolase domain containing 10	Cytoplasm	enzyme	
−26.16	8.26E-03	ENSG00000165660		ABRAXAS2	abraxas 2, BRISC complex subunit	Nucleus	other	
−27.92	5.54E-03	ENSG00000226359		ACTG1P24	actin gamma 1 pseudogene 24	Other	other	
−23.18	5.24E-06	ENSG00000168594		ADAM29	ADAM metallopeptidase domain 29	Plasma Membrane	peptidase	
−26.19	7.91E-03	ENSG00000274376		ADAMTS7P1	ADAMTS7 pseudogene 1	Other	other	
−16.27	7.38E-02	ENSG00000065457		ADAT1	adenosine deaminase, tRNA specific 1	Other	enzyme	
26.85	7.20E-03	ENSG00000204818		ADGRF5P2	adhesion G protein-coupled receptor F5 pseudogene 2	Other	other	
25.98	8.69E-03	ENSG00000156920		ADGRG4	adhesion G protein-coupled receptor G4	Plasma Membrane	G-protein coupled receptor	
−28.07	5.54E-03	ENSG00000243289		AGAP13P	ArfGAP with GTPase domain, ankyrin repeat and PH domain 13, pseudogene	Other	other	
30.00	4.30E-03	ENSG00000196581		AJAP1	adherens junctions associated protein 1	Plasma Membrane	other	
26.13	8.21E-03	ENSG00000140057		AK7	adenylate kinase 7	Cytoplasm	kinase	
−29.86	4.30E-03	ENSG00000108602		ALDH3A1	aldehyde dehydrogenase 3 family member A1	Cytoplasm	enzyme	
−26.69	7.65E-03	ENSG00000163046		ANKRD30BL	ankyrin repeat domain 30B like	Other	other	
−26.36	7.91E-03	ENSG00000224309		ANKRD30BP2	ankyrin repeat domain 30B pseudogene 2	Other	other	
29.96	4.30E-03	ENSG00000184945		AQP12A/AQP12B	aquaporin 12A	Cytoplasm	transporter	
30.00	4.30E-03	ENSG00000198576		ARC	activity regulated cytoskeleton associated protein	Cytoplasm	other	
25.98	8.69E-03	ENSG00000127249		ATP13A4	ATPase 13A4	Cytoplasm	transporter	
27.73	5.66E-03	ENSG00000228847		ATP5G2P4	ATP synthase, H+ transporting, mitochondrial Fo complex subunit C2 (subunit 9) pseudogene 4	Other	other	
20.55	9.40E-02	ENSG00000230223		ATXN8OS	ATXN8 opposite strand (non-protein coding)	Other	other	
27.05	7.01E-03	ENSG00000148090		AUH	AU RNA binding methylglutaconyl-CoA hydratase	Cytoplasm	enzyme	
−29.79	4.30E-03	ENSG00000198049		AVPR1B	arginine vasopressin receptor 1B	Plasma Membrane	G-protein coupled receptor	AVP, lypressin
−29.84	4.30E-03	ENSG00000187172		BAGE2	BAGE family member 2	Other	other	
−25.63	1.10E-02	ENSG00000100739		BDKRB1	bradykinin receptor B1	Plasma Membrane	G-protein coupled receptor	
−27.71	5.66E-03	ENSG00000224809		BEND3P2	BEN domain containing 3 pseudogene 2	Other	other	
25.69	9.93E-03	ENSG00000204960		BLACE	B-cell acute lymphoblastic leukemia expressed	Other	other	
−29.71	4.30E-03	ENSG00000186222		BLOC1S4	biogenesis of lysosomal organelles complex 1 subunit 4	Cytoplasm	other	
−29.92	4.30E-03	ENSG00000125845		BMP2	bone morphogenetic protein 2	Extracellular Space	growth factor	
−26.19	7.91E-03	ENSG00000198183		BPIFA1	BPI fold containing family A member 1	Extracellular Space	other	
28.23	5.54E-03	ENSG00000224775		BRAFP1	BRAF pseudogene 1	Other	other	
−29.84	4.30E-03	ENSG00000226913		BSN-AS2	BSN antisense RNA 2 (head to head)	Other	other	
−30.00	4.30E-03	ENSG00000137656		BUD13	BUD13 homolog	Nucleus	other	
−26.19	7.91E-03	ENSG00000173237		C11orf86	chromosome 11 open reading frame 86	Other	other	
−28.54	5.52E-03	ENSG00000157330		C1orf158	chromosome 1 open reading frame 158	Other	other	
−25.71	9.87E-03	ENSG00000188511		C22orf34	chromosome 22 open reading frame 34	Other	other	
−26.19	7.91E-03	ENSG00000173557		C2orf70	chromosome 2 open reading frame 70	Nucleus	other	
−30.00	4.30E-03	ENSG00000237787		C3orf79	chromosome 3 open reading frame 79	Other	other	
26.46	7.91E-03	ENSG00000082213		C5orf22	chromosome 5 open reading frame 22	Other	other	
2.65	6.09E-02	ENSG00000256462		CALML3	calmodulin like 3	Cytoplasm	other	
−26.19	7.91E-03	ENSG00000141668		CBLN2	cerebellin 2 precursor	Extracellular Space	other	
−26.49	7.91E-03	ENSG00000183287		CCBE1	collagen and calcium binding EGF domains 1	Extracellular Space	other	
−28.54	5.52E-03	ENSG00000100147		CCDC134	coiled-coil domain containing 134	Other	other	
28.23	5.54E-03	ENSG00000163081		CCDC140	coiled-coil domain containing 140	Other	other	
−28.49	5.52E-03	ENSG00000248712		CCDC153	coiled-coil domain containing 153	Other	other	
−28.00	5.54E-03	ENSG00000276409		CCL14	C–C motif chemokine ligand 14	Extracellular Space	cytokine	
−29.96	4.30E-03	ENSG00000133101		CCNA1	cyclin A1	Nucleus	other	
−28.80	5.52E-03	ENSG00000238241		CCR12P	C–C motif chemokine receptor 12, pseudogene	Other	other	
−26.36	7.91E-03	ENSG00000204933		CD177P1	CD177 molecule pseudogene 1	Other	other	
−27.06	6.99E-03	ENSG00000158477		CD1A	CD1a molecule	Plasma Membrane	other	
−26.69	7.65E-03	ENSG00000184661		CDCA2	cell division cycle associated 2	Nucleus	other	
−27.06	6.99E-03	ENSG00000145526		CDH18	cadherin 18	Plasma Membrane	other	
17.96	2.14E-03	ENSG00000113100		CDH9	cadherin 9	Plasma Membrane	other	
26.76	7.20E-03	ENSG00000170312		CDK1	cyclin dependent kinase 1	Nucleus	kinase	dinaciclib, AG 024322, AZD5438, milciclib, SB-1317, roniciclib, alvocidib
−25.71	9.87E-03	ENSG00000230666		CEACAM22P	carcinoembryonic antigen related cell adhesion molecule 22, pseudogene	Other	other	
−26.19	7.91E-03	ENSG00000273203		CECR7	cat eye syndrome chromosome region, candidate 7 (non-protein coding)	Other	other	
19.24	6.08E-02	ENSG00000267448		CELF5	CUGBP Elav-like family member 5	Nucleus	other	
25.98	8.69E-03	ENSG00000272514		CFAP206	cilia and flagella associated protein 206	Cytoplasm	other	
−27.02	7.01E-03	ENSG00000258469		CHMP4BP1	charged multivesicular body protein 4B pseudogene 1	Other	other	
−14.77	7.91E-03	ENSG00000181072		CHRM2	cholinergic receptor muscarinic 2	Plasma Membrane	G-protein coupled receptor	fesoterodine, ABT-089, dimetindene, protoveratrine derivative, aclidinium, dextromethorphan/quinidine, umeclidinium, glycopyrrolate/indacaterol, umeclidinium/vilanterol, olodaterol/tiotropium, indacaterol/tiotropium, formoterol/glycopyrrolate, donepezil/solifenacin, glycopyrrolate/indacaterol/mometasone furoate, atropine/edrophonium, cyclopentolate/phenylephrine, albuterol/ipratropium, trihexyphenidyl, carbamylcholine, diphenhydramine/ibuprofen, methacholine, dexamethasone/olanzapine, diphenhydramine, doxepin, quinidine, procyclidine, trospium, rocuronium, homatropine, dicyclomine, methantheline, orphenadrine, fluoxetine/olanzapine, doxacurium, aspirin/caffeine/orphenadrine, propantheline, tridihexethyl, biperiden, anisotropine methylbromide, glycopyrrolate, atropine/hyoscyamine/phenobarbital/scopolamine, atropine/diphenoxylate, pipecuronium, flavoxate, chlorpheniramine/methscopolamine/phenylephrine, mepenzolic acid, homatropine methylbromide, hydroxyamphetamine/tropicamide, cisatracurium, hyoscyamine/phenobarbital, triflupromazine, bethanechol, olanzapine, oxybutynin, tropicamide, solifenacin, cyclopentolate, gallamine triethiodide, tolterodine, cevimeline, acetylcholine, ipratropium, atropine, pilocarpine, benztropine, hyoscyamine, arecoline, scopolamine, N-methylscopolamine, tiotropium, carbinoxamine, buclizine, benzquinamide, diphenhydramine/phenylephrine, brompheniramine
−27.92	5.54E-03	ENSG00000172322		CLEC12A	C-type lectin domain family 12 member A	Plasma Membrane	other	
−28.07	5.54E-03	ENSG00000111729		CLEC4A	C-type lectin domain family 4 member A	Plasma Membrane	transmembrane receptor	
−30.00	4.30E-03	ENSG00000104938		CLEC4M	C-type lectin domain family 4 member M	Plasma Membrane	other	
−25.71	9.87E-03	ENSG00000205057		CLLU1OS	chronic lymphocytic leukemia up-regulated 1 opposite strand	Other	other	
26.50	7.91E-03	ENSG00000155052		CNTNAP5	contactin associated protein like 5	Other	other	
−27.92	5.54E-03	ENSG00000231595		COX10	COX10, heme A:farnesyltransferase cytochrome c oxidase assembly factor	Cytoplasm	enzyme	
−27.42	5.67E-03	ENSG00000258981		COX5AP2	cytochrome c oxidase subunit 5A pseudogene 2	Other	other	
29.36	4.56E-03	ENSG00000117322		CR2	complement C3d receptor 2	Plasma Membrane	transmembrane receptor	
−16.35	5.28E-02	ENSG00000276076		CRYAA/LOC102724652	crystallin alpha A	Cytoplasm	other	
28.23	5.54E-03	ENSG00000135222		CSN2	casein beta	Extracellular Space	kinase	
−29.34	4.45E-03	ENSG00000170367		CST5	cystatin D	Cytoplasm	other	
−29.44	4.37E-03	ENSG00000228836		CT45A10/CT45A5	cancer/testis antigen family 45 member A5	Other	other	
−26.69	7.65E-03	ENSG00000235857		CTBP2P1	C-terminal binding protein 2 pseudogene 1	Other	other	
−26.36	7.91E-03	ENSG00000234993		CUBNP2	cubilin pseudogene 2	Other	other	
−25.71	9.87E-03	ENSG00000120280		CXorf21	chromosome X open reading frame 21	Other	enzyme	
−26.19	7.91E-03	ENSG00000183035		CYLC1	cylicin 1	Cytoplasm	other	
27.73	5.66E-03	ENSG00000155833		CYLC2	cylicin 2	Other	other	
−26.19	7.91E-03	ENSG00000036530		CYP46A1	cytochrome P450 family 46 subfamily A member 1	Cytoplasm	enzyme	
−27.43	5.90E-03	ENSG00000173406		DAB1	DAB1, reelin adaptor protein	Cytoplasm	other	
−26.36	7.91E-03	ENSG00000179284		DAND5	DAN domain BMP antagonist family member 5	Extracellular Space	other	
−26.19	7.91E-03	ENSG00000236709		DAPK1-IT1	DAPK1 intronic transcript 1	Other	other	
30.00	4.30E-03	ENSG00000152670		DDX4	DEAD-box helicase 4	Nucleus	enzyme	
27.24	6.68E-03	ENSG00000164822		DEFA6	defensin alpha 6	Extracellular Space	other	
−29.77	4.30E-03	ENSG00000205883		DEFB135	defensin beta 135	Other	other	
26.47	7.91E-03	ENSG00000058866		DGKG	diacylglycerol kinase gamma	Cytoplasm	kinase	
−26.36	7.91E-03	ENSG00000104808		DHDH	dihydrodiol dehydrogenase	Other	enzyme	
−17.70	6.24E-02	ENSG00000187908		DMBT1	deleted in malignant brain tumors 1	Plasma Membrane	transmembrane receptor	
−26.19	7.91E-03	ENSG00000157851		DPYSL5	dihydropyrimidinase like 5	Cytoplasm	enzyme	
−25.71	9.87E-03	ENSG00000125821		DTD1	D-tyrosyl-tRNA deacylase 1	Cytoplasm	enzyme	
25.98	8.69E-03	ENSG00000198842		DUSP27	dual specificity phosphatase 27, atypical	Other	phosphatase	
−30.00	4.30E-03	ENSG00000229758		DYNLT3P2	dynein light chain Tctex-type 3 pseudogene 2	Other	other	
−22.51	3.61E-02	ENSG00000104823		ECH1	enoyl-CoA hydratase 1	Cytoplasm	enzyme	
−28.07	5.54E-03	ENSG00000268222		EEF1A1P7	eukaryotic translation elongation factor 1 alpha 1 pseudogene 7	Other	other	
−28.80	5.52E-03	ENSG00000235549		EIF1P2	eukaryotic translation initiation factor 1 pseudogene 2	Other	other	
−25.71	9.87E-03	ENSG00000227295		ELL2P1	elongation factor for RNA polymerase II 2 pseudogene 1	Other	other	
−25.63	1.10E-02	ENSG00000144488		ESPNL	espin like	Extracellular Space	other	
−28.07	5.54E-03	ENSG00000253831		ETV3L	ETS variant 3 like	Other	other	
28.19	5.52E-03	ENSG00000231882		F10-AS1	F10 antisense RNA 1	Other	other	
−26.19	7.91E-03	ENSG00000226766		FABP7P1	fatty acid binding protein 7 pseudogene 1	Other	other	
−26.96	7.09E-03	ENSG00000183508		FAM46C	family with sequence similarity 46 member C	Extracellular Space	other	
30.00	4.30E-03	ENSG00000174678		FAM47DP	family with sequence similarity 47 member D, pseudogene	Other	other	
−27.40	6.75E-03	ENSG00000197872		FAM49A	family with sequence similarity 49 member A	Other	other	
26.50	7.91E-03	ENSG00000253488		FAM60DP	family with sequence similarity 60, member A pseudogene	Other	other	
26.47	7.91E-03	ENSG00000224710	D	FAM90A26 (includes others)	family with sequence similarity 90, member A26, pseudogene	Other	other	
26.47	7.91E-03	ENSG00000231656	D	FAM90A26 (includes others)	family with sequence similarity 90, member A26, pseudogene	Other	other	
26.47	7.91E-03	ENSG00000237122	D	FAM90A26 (includes others)	family with sequence similarity 90, member A26, pseudogene	Other	other	
−22.53	4.68E-02	ENSG00000231060		FARSBP1	phenylalanyl-tRNA synthetase beta subunit pseudogene 1	Other	other	
29.24	4.62E-03	ENSG00000132185		FCRLA	Fc receptor like A	Plasma Membrane	other	
19.89	5.10E-02	ENSG00000181171		FER1L6-AS1	FER1L6 antisense RNA 1	Other	other	
30.00	4.30E-03	ENSG00000146618		FERD3L	Fer3 like bHLH transcription factor	Nucleus	transcription regulator	
28.56	5.52E-03	ENSG00000106692		FKTN	fukutin	Extracellular Space	other	
−25.71	9.87E-03	ENSG00000257800		FNBP1P1	formin binding protein 1 pseudogene 1	Other	other	
26.47	7.91E-03	ENSG00000086205		FOLH1	folate hydrolase 1	Plasma Membrane	peptidase	capromab pendetide
−6.35	7.87E-02	ENSG00000110195		FOLR1	folate receptor 1	Plasma Membrane	transporter	farletuzumab
−26.96	7.09E-03	ENSG00000258421		FXNP1	frataxin pseudogene 1	Other	other	
27.73	5.66E-03	ENSG00000177736		FXNP2	frataxin pseuodgene 2	Other	other	
26.13	8.21E-03	ENSG00000136750		GAD2	glutamate decarboxylase 2	Cytoplasm	enzyme	valproic acid
26.50	7.91E-03	ENSG00000257594		GALNT4	polypeptide N-acetylgalactosaminyltransferase 4	Cytoplasm	enzyme	
25.98	8.69E-03	ENSG00000109534		GAR1	GAR1 ribonucleoprotein	Nucleus	other	
30.00	4.30E-03	ENSG00000228376		GAS2L1P2	growth arrest specific 2 like 1 pseudogene 2	Other	other	
−25.63	1.10E-02	ENSG00000272695		GAS6-AS2	GAS6 antisense RNA 2 (head to head)	Other	other	
26.85	7.20E-03	ENSG00000168546		GFRA2	GDNF family receptor alpha 2	Plasma Membrane	transmembrane receptor	
−26.19	7.91E-03	ENSG00000125861		GFRA4	GDNF family receptor alpha 4	Plasma Membrane	transmembrane receptor	
−29.72	4.37E-03	ENSG00000197421		GGT3P	gamma-glutamyltransferase 3 pseudogene	Extracellular Space	other	
−28.54	5.52E-03	ENSG00000223893		GNL2P1	G protein nucleolar 2 pseudogene 1	Other	other	
−27.06	6.99E-03	ENSG00000111711		GOLT1B	golgi transport 1B	Cytoplasm	other	
−27.02	7.01E-03	ENSG00000235984		GPC5-AS1	GPC5 antisense RNA 1	Other	other	
28.58	5.52E-03	ENSG00000232885		GPC5-AS2	GPC5 antisense RNA 2	Other	other	
−26.36	7.91E-03	ENSG00000182771		GRID1	glutamate ionotropic receptor delta type subunit 1	Plasma Membrane	ion channel	
27.43	6.04E-03	ENSG00000140307		GTF2A2	general transcription factor IIA subunit 2	Nucleus	transcription regulator	
−26.19	7.91E-03	ENSG00000153767		GTF2E1	general transcription factor IIE subunit 1	Nucleus	transcription regulator	
−29.44	4.45E-03	ENSG00000237099		GYG1P2	glycogenin 1 pseudogene 2	Other	other	
21.02	7.22E-02	ENSG00000145649		GZMA	granzyme A	Cytoplasm	peptidase	
28.14	5.52E-03	ENSG00000270604		HCG17	HLA complex group 17 (non-protein coding)	Other	other	
−30.00	4.30E-03	ENSG00000164588		HCN1	hyperpolarization activated cyclic nucleotide gated potassium channel 1	Plasma Membrane	ion channel	
26.47	7.91E-03	ENSG00000281831		HCP5B	HLA complex P5B (non-protein coding)	Other	other	
−28.32	5.52E-03	ENSG00000113924		HGD	homogentisate 1,2-dioxygenase	Cytoplasm	enzyme	
28.23	5.52E-03	ENSG00000273703		HIST1H2BM	histone cluster 1H2B family member m	Nucleus	other	
−29.72	2.14E-03	ENSG00000274641		HIST1H2BO	histone cluster 1H2B family member o	Nucleus	other	
−30.00	4.30E-03	ENSG00000224557		HLA-DPB2	major histocompatibility complex, class II, DP beta 2 (pseudogene)	Other	other	
−30.00	4.30E-03	ENSG00000232629		HLA-DQB2	major histocompatibility complex, class II, DQ beta 2	Plasma Membrane	transmembrane receptor	
28.15	5.54E-03	ENSG00000220557		HMGB1P13	high mobility group box 1 pseudogene 13	Other	other	
28.15	5.54E-03	ENSG00000253516		HMGB1P41	high mobility group box 1 pseudogene 41	Other	other	
−28.07	5.54E-03	ENSG00000237285		HNRNPA1P2	heterogeneous nuclear ribonucleoprotein A1 pseudogene 2	Other	other	
29.41	4.37E-03	ENSG00000249271		HNRNPA1P44	heterogeneous nuclear ribonucleoprotein A1 pseudogene 44	Other	other	
−26.80	7.20E-03	ENSG00000120075		HOXB5	homeobox B5	Nucleus	transcription regulator	
−29.82	4.30E-03	ENSG00000123407		HOXC12	homeobox C12	Nucleus	transcription regulator	
29.24	4.62E-03	ENSG00000223855		HRAT92	heart tissue-associated transcript 92	Other	other	
−26.19	7.91E-03	ENSG00000248159		HSPA8P11	heat shock protein family A (Hsp70) member 8 pseudogene 11	Other	other	
26.13	8.21E-03	ENSG00000250356		HSPE1P23	heat shock protein family E (Hsp10) member 1 pseudogene 23	Other	other	
25.69	9.93E-03	ENSG00000232015		HSPE1P25	heat shock protein family E (Hsp10) member 1 pseudogene 25	Other	other	
−27.92	5.54E-03	ENSG00000148680		HTR7	5-hydroxytryptamine receptor 7	Plasma Membrane	G-protein coupled receptor	iloperidone, asenapine, sultopride, lurasidone, vortioxetine, brexpiprazole, eletriptan, almotriptan, amoxapine, fenfluramine, methysergide, ergotamine
30.00	4.30E-03	ENSG00000121351		IAPP	islet amyloid polypeptide	Extracellular Space	other	
−29.81	4.30E-03	ENSG00000137965		IFI44	interferon induced protein 44	Cytoplasm	other	
−26.19	7.91E-03	ENSG00000272311		IFNL4P1	interferon lambda 4 pseudogene 1	Other	other	
−30.00	4.30E-03	ENSG00000163395		IGFN1	immunoglobulin-like and fibronectin type III domain containing 1	Nucleus	other	
26.13	8.21E-03	ENSG00000239855		IGKV1-6	immunoglobulin kappa variable 1-6	Other	other	
−29.75	4.30E-03	ENSG00000253823		IGLV1-62	immunoglobulin lambda variable 1–62 (pseudogene)	Other	other	
25.98	8.69E-03	ENSG00000253889		IGLVI-38	immunoglobulin lambda variable (I)-38 (pseudogene)	Other	other	
−26.36	7.91E-03	ENSG00000196083		IL1RAP	interleukin 1 receptor accessory protein	Plasma Membrane	transmembrane receptor	CAN04
−25.71	9.87E-03	ENSG00000162594		IL23R	interleukin 23 receptor	Plasma Membrane	transmembrane receptor	
28.23	5.54E-03	ENSG00000217539		IQCB2P	IQ motif containing B2 pseudogene	Other	other	
25.98	8.69E-03	ENSG00000255679		JRKL-AS1	JRKL antisense RNA 1	Other	other	
−15.26	8.56E-03	ENSG00000242370		KCNAB1-AS1	KCNAB1 antisense RNA 1	Other	other	
27.58	5.67E-03	ENSG00000280650		KCNIP4-IT1	KCNIP4 intronic transcript 1	Other	other	
−15.90	5.44E-03	ENSG00000162989		KCNJ3	potassium voltage-gated channel subfamily J member 3	Plasma Membrane	ion channel	
−27.92	5.54E-03	ENSG00000257069		KCNK4-TEX40	KCNK4-TEX40 readthrough	Other	other	
−25.63	1.10E-02	ENSG00000143603		KCNN3	potassium calcium-activated channel subfamily N member 3	Plasma Membrane	ion channel	
−22.96	3.92E-02	ENSG00000150361		KLHL1	kelch like family member 1	Cytoplasm	other	
27.01	6.99E-03	ENSG00000250221		KRT8P32	keratin 8 pseudogene 32	Other	other	
27.58	5.67E-03	ENSG00000198841		KTI12	KTI12 chromatin associated homolog	Other	other	
−27.02	7.01E-03	ENSG00000217783		LDHAL6FP	lactate dehydrogenase A like 6F, pseudogene	Other	other	
28.32	5.52E-03	ENSG00000179676		LINC00305	long intergenic non-protein coding RNA 305	Other	other	
19.43	1.87E-02	ENSG00000226250		LINC00408	long intergenic non-protein coding RNA 408	Other	other	
−26.97	7.01E-03	ENSG00000236384		LINC00479	long intergenic non-protein coding RNA 479	Plasma Membrane	other	
−25.71	9.87E-03	ENSG00000251533		LINC00605	long intergenic non-protein coding RNA 605	Other	other	
25.98	8.69E-03	ENSG00000233746		LINC00656	long intergenic non-protein coding RNA 656	Other	other	
−27.92	5.54E-03	ENSG00000229404		LINC00858	long intergenic non-protein coding RNA 858	Other	other	
−14.31	1.94E-02	ENSG00000214145		LINC00887	long intergenic non-protein coding RNA 887	Other	other	
−26.19	7.91E-03	ENSG00000259361		LINC00927	long intergenic non-protein coding RNA 927	Other	other	
−26.36	7.91E-03	ENSG00000230174		LINC01149	long intergenic non-protein coding RNA 1149	Other	other	
−26.36	7.91E-03	ENSG00000274827		LINC01297		Other	other	
−26.19	7.91E-03	ENSG00000244578		LINC01391	long intergenic non-protein coding RNA 1391	Other	other	
−30.00	4.30E-03	ENSG00000249306		LINC01411	long intergenic non-protein coding RNA 1411	Other	other	
26.47	7.91E-03	ENSG00000254211		LINC01485	long intergenic non-protein coding RNA 1485	Other	other	
−27.43	5.67E-03	ENSG00000231422		LINC01516	long intergenic non-protein coding RNA 1516	Other	other	
27.58	5.67E-03	ENSG00000262468		LINC01569	long intergenic non-protein coding RNA 1569	Other	other	
−27.73	5.54E-03	ENSG00000227115		LINC01630	long intergenic non-protein coding RNA 1630	Other	other	
−26.36	7.91E-03	ENSG00000230768		LINC01676	long intergenic non-protein coding RNA 1676	Other	other	
30.00	4.30E-03	ENSG00000227181		LINC01688	long intergenic non-protein coding RNA 1688	Other	other	
−28.07	5.54E-03	ENSG00000232046		LINC01798	long intergenic non-protein coding RNA 1798	Other	other	
−30.00	1.44E-03	ENSG00000223466		LINC01825		Other	other	
−28.07	5.54E-03	ENSG00000264345		LINC01894		Other	other	
−25.63	1.10E-02	ENSG00000254299		LINC01944		Other	other	
27.86	5.54E-03	ENSG00000251085		LINC01969	long intergenic non-protein coding RNA 1969	Other	other	
−29.98	4.30E-03	ENSG00000273033		LINC02035	long intergenic non-protein coding RNA 2035	Other	other	
−29.92	4.30E-03	ENSG00000229155		LINC02038		Other	other	
−29.97	4.30E-03	ENSG00000241696		LINC02053	long intergenic non-protein coding RNA 2053	Other	other	
−30.00	4.30E-03	ENSG00000260896		LINC02170	long intergenic non-protein coding RNA 2170	Other	other	
29.90	4.30E-03	ENSG00000260792		LINC02280	long intergenic non-protein coding RNA 2280	Other	other	
−28.54	5.52E-03	ENSG00000257056		LINC02282	long intergenic non-protein coding RNA 2282	Other	other	
29.96	4.30E-03	ENSG00000258859		LINC02296		Other	other	
28.23	5.52E-03	ENSG00000258998		LINC02302	long intergenic non-protein coding RNA 2302	Other	other	
−18.02	6.20E-02	ENSG00000255002		LINC02324	long intergenic non-protein coding RNA 2324	Other	other	
29.60	4.37E-03	ENSG00000248338		LINC02472		Other	other	
27.73	5.66E-03	ENSG00000249362		LINC02488		Other	other	
−27.87	5.54E-03	ENSG00000168216		LMBRD1	LMBR1 domain containing 1	Cytoplasm	transporter	
−25.63	1.10E-02	ENSG00000257711		LOC100505978	uncharacterized LOC100505978	Other	other	
28.23	5.52E-03	ENSG00000228877		LOC100506532	uncharacterized LOC100506532	Other	other	
−28.52	5.52E-03	ENSG00000243491		LOC100996549	uncharacterized LOC100996549	Other	other	
30.00	4.30E-03	ENSG00000225768		LOC101927523	uncharacterized LOC101927523	Other	other	
−30.00	4.30E-03	ENSG00000236039		LOC101927630	uncharacterized LOC101927630	Other	other	
−26.19	7.91E-03	ENSG00000249419		LOC101928052	uncharacterized LOC101928052	Other	other	
−26.36	7.91E-03	ENSG00000264464		LOC101928144	uncharacterized LOC101928144	Other	other	
−29.63	4.45E-03	ENSG00000253447		LOC102724551	uncharacterized LOC102724551	Other	other	
−26.36	7.91E-03	ENSG00000259170		LOC104613533	uncharacterized LOC104613533	Other	other	
−28.49	5.52E-03	ENSG00000257023		LOC105369691	uncharacterized LOC105369691	Other	other	
20.58	8.43E-02	ENSG00000258167		LOC105369736	uncharacterized LOC105369736	Other	other	
−29.77	4.30E-03	ENSG00000269720		LOC105372343		Other	other	
30.00	4.30E-03	ENSG00000273877		LOC105373373		Other	other	
−27.02	7.01E-03	ENSG00000251652		LOC105374344		Other	other	
−26.19	7.91E-03	ENSG00000275846		LOC105374989	uncharacterized LOC105374989	Other	other	
29.93	4.30E-03	ENSG00000253699		LOC105375623	uncharacterized LOC105375623	Other	other	
−17.61	1.59E-03	ENSG00000253477		LOC105375690	uncharacterized LOC105375690	Other	other	
27.81	5.54E-03	ENSG00000267559		LOC105376108		Other	other	
−27.32	6.26E-03	ENSG00000254670		LOC105376557	uncharacterized LOC105376557	Other	other	
−26.36	7.91E-03	ENSG00000226969		LOC105378591		Other	other	
29.38	4.45E-03	ENSG00000251616		LOC105379178	uncharacterized LOC105379178	Other	other	
−26.36	7.91E-03	ENSG00000259697		LOC107984784	uncharacterized LOC107984784	Other	other	
−26.36	7.91E-03	ENSG00000255545		LOC283177	uncharacterized LOC283177	Other	other	
30.00	4.30E-03	ENSG00000250658		LOC339975	uncharacterized LOC339975	Other	other	
−29.97	2.89E-04	ENSG00000243440		LOC388813	uncharacterized protein ENSP00000383407-like	Other	other	
−28.54	5.52E-03	ENSG00000263711		LOC400655	uncharacterized LOC400655	Other	other	
28.19	5.52E-03	ENSG00000277654		LOC440311	NOP53 ribosome biogenesis factor pseudogene	Other	other	
28.23	5.54E-03	ENSG00000259663		LOC642366	uncharacterized LOC642366	Other	other	
26.13	8.21E-03	ENSG00000278849		LOC642929	general transcription factor II, i pseudogene	Other	other	
−29.62	4.37E-03	ENSG00000230563		LOC643406	uncharacterized LOC643406	Other	other	
−26.19	7.91E-03	ENSG00000241014		LOC653160	uncharacterized LOC653160	Other	other	
30.00	4.30E-03	ENSG00000204913		LRRC3C	leucine rich repeat containing 3C	Other	other	
−30.00	4.30E-03	ENSG00000141639		MAPK4	mitogen-activated protein kinase 4	Cytoplasm	kinase	
−28.54	5.52E-03	ENSG00000144583		4-Mar	membrane associated ring–CH–type finger 4	Cytoplasm	enzyme	
−29.97	4.30E-03	ENSG00000248109		MARCOL		Other	other	
−26.19	7.91E-03	ENSG00000139915		MDGA2	MAM domain containing glycosylphosphatidylinositol anchor 2	Other	other	
−25.71	9.87E-03	ENSG00000130772		MED18	mediator complex subunit 18	Nucleus	other	
−29.82	4.30E-03	ENSG00000229623		METTL21AP1	methyltransferase like 21A pseudogene 1	Other	other	
−26.19	7.91E-03	ENSG00000250878		METTL21EP	methyltransferase like 21E, pseudogene	Other	other	
26.13	8.21E-03	ENSG00000100060		MFNG	MFNG O-fucosylpeptide 3-beta-N-acetylglucosaminyltransferase	Cytoplasm	enzyme	
30.00	4.30E-03	ENSG00000116691		MIIP	migration and invasion inhibitory protein	Other	other	
−25.71	9.87E-03	ENSG00000207779		mir-15	microRNA 15a	Cytoplasm	microRNA	
−28.33	5.52E-03	ENSG00000265322	D	mir-3118	microRNA 3118-3	Other	microRNA	
−28.33	5.52E-03	ENSG00000265793	D	mir-3118	microRNA 3118-3	Other	microRNA	
28.15	5.54E-03	ENSG00000266299	D	mir-3118	microRNA 3118-3	Other	microRNA	
27.01	6.99E-03	ENSG00000265932		mir-3146	microRNA 3146	Cytoplasm	microRNA	
26.85	7.20E-03	ENSG00000212014		mir-506	microRNA 508	Cytoplasm	microRNA	
−28.42	5.52E-03	ENSG00000215973		mir-933	microRNA 933	Cytoplasm	microRNA	
27.01	6.99E-03	ENSG00000264049		MIR4737	microRNA 4737	Cytoplasm	microRNA	
−28.07	5.54E-03	ENSG00000278108		MIR6757	microRNA 6757	Other	microRNA	
−26.36	7.91E-03	ENSG00000224430		MKRN5P	makorin ring finger protein 5, pseudogene	Other	other	
−29.52	4.37E-03	ENSG00000230500		MKX-AS1	MKX antisense RNA 1	Other	other	
−27.06	6.99E-03	ENSG00000082515		MRPL22	mitochondrial ribosomal protein L22	Cytoplasm	other	
26.85	7.20E-03	ENSG00000257927		MRPS36P5	mitochondrial ribosomal protein S36 pseudogene 5	Other	other	
−15.27	9.86E-02	ENSG00000135972		MRPS9	mitochondrial ribosomal protein S9	Cytoplasm	other	
26.50	7.91E-03	ENSG00000120149		MSX2	msh homeobox 2	Nucleus	transcription regulator	
25.98	8.69E-03	ENSG00000227035		MTATP6P18		Other	other	
27.58	5.67E-03	ENSG00000237055		MTCO1P48		Other	other	
27.80	5.54E-03	ENSG00000236211		MTCO1P7		Other	other	
19.32	5.83E-02	ENSG00000233888		MTCO2P17		Other	other	
19.32	4.66E-02	ENSG00000231576		MTCO2P20		Other	other	
−26.36	7.91E-03	ENSG00000254118		MTCYBP20		Other	other	
−26.19	7.91E-03	ENSG00000263241		MTCYBP33		Other	other	
−28.80	5.52E-03	ENSG00000271687		MTND5P10	mitochondrially encoded NADH:ubiquinone oxidoreductase core subunit 5 pseudogene 10	Other	other	
−26.36	7.91E-03	ENSG00000256045		MTRNR2L10	MT-RNR2-like 10	Other	other	
−23.86	5.67E-03	ENSG00000177034		MTX3	metaxin 3	Other	other	
−30.00	4.30E-03	ENSG00000169876		MUC17	mucin 17, cell surface associated	Plasma Membrane	other	
−27.92	5.54E-03	ENSG00000111046		MYF6	myogenic factor 6	Nucleus	transcription regulator	
−25.63	1.10E-02	ENSG00000187616		MYMK	myomaker, myoblast fusion factor	Plasma Membrane	other	
−26.36	7.91E-03	ENSG00000225619		MYT1L-AS1	MYT1L antisense RNA 1	Other	other	
−29.47	4.39E-03	ENSG00000204272		NBDY	negative regulator of P-body association	Other	other	
26.13	8.21E-03	ENSG00000124479		NDP	NDP, norrin cystine knot growth factor	Extracellular Space	growth factor	
−29.85	4.30E-03	ENSG00000166507		NDST2	N-deacetylase and N-sulfotransferase 2	Cytoplasm	enzyme	
−29.94	4.30E-03	ENSG00000242943		NKAIN1P1	sodium/potassium transporting ATPase interacting 1 pseudogene 1	Other	other	
26.83	7.14E-03	ENSG00000248745		NMNAT1P4	NMNAT1 pseudogene 4	Other	other	
−29.59	4.34E-03	ENSG00000171596		NMUR1	neuromedin U receptor 1	Plasma Membrane	G-protein coupled receptor	
−17.20	3.35E-02	ENSG00000086991		NOX4	NADPH oxidase 4	Cytoplasm	enzyme	imipramine blue
−28.00	5.54E-03	ENSG00000112333		NR2E1	nuclear receptor subfamily 2 group E member 1	Nucleus	ligand-dependent nuclear receptor	
−29.84	4.30E-03	ENSG00000225964		NRIR		Other	other	
−15.04	4.30E-03	ENSG00000101188		NTSR1	neurotensin receptor 1	Plasma Membrane	G-protein coupled receptor	contulakin-G
−22.99	5.52E-03	ENSG00000152463		OLAH	oleoyl-ACP hydrolase	Cytoplasm	enzyme	
27.58	5.67E-03	ENSG00000125510		OPRL1	opioid related nociceptin receptor 1	Plasma Membrane	G-protein coupled receptor	ZP120, buprenorphine/naloxone, cebranopadol, buprenorphine
27.83	5.54E-03	ENSG00000171199		OPRPN	opiorphin prepropeptide	Extracellular Space	other	
−28.55	5.52E-03	ENSG00000180409		OR10AA1P	olfactory receptor family 10 subfamily AA member 1 pseudogene	Other	other	
26.85	7.20E-03	ENSG00000130538	D	OR11H12 (includes others)	olfactory receptor family 11 subfamily H member 12	Plasma Membrane	other	
26.50	7.91E-03	ENSG00000257115	D	OR11H12 (includes others)	olfactory receptor family 11 subfamily H member 12	Plasma Membrane	other	
26.50	7.91E-03	ENSG00000203581		OR1F2P	olfactory receptor family 1 subfamily F member 2 pseudogene	Other	other	
27.23	6.68E-03	ENSG00000239967		OR2A41P	olfactory receptor family 2 subfamily A member 41 pseudogene	Other	other	
−27.42	6.04E-03	ENSG00000141194		OR4D1	olfactory receptor family 4 subfamily D member 1	Plasma Membrane	G-protein coupled receptor	
27.48	5.96E-03	ENSG00000254430		OR6M3P	olfactory receptor family 6 subfamily M member 3 pseudogene	Other	other	
−26.19	7.91E-03	ENSG00000197125		OR8B8	olfactory receptor family 8 subfamily B member 8	Plasma Membrane	G-protein coupled receptor	
27.99	5.54E-03	ENSG00000229386		OR8B9P	olfactory receptor family 8 subfamily B member 9 pseudogene	Other	other	
26.47	7.91E-03	ENSG00000186508		OR9I2P	olfactory receptor family 9 subfamily I member 2 pseudogene	Other	other	
−25.63	1.10E-02	ENSG00000234315		OSTCP5	oligosaccharyltransferase complex subunit pseudogene 5	Other	other	
30.00	4.30E-03	ENSG00000204237		OXLD1	oxidoreductase like domain containing 1	Cytoplasm	other	
26.13	8.21E-03	ENSG00000225638		PABPC1P12	poly(A) binding protein cytoplasmic 1 pseudogene 12	Other	other	
−28.29	5.52E-03	ENSG00000158006		PAFAH2	platelet activating factor acetylhydrolase 2	Cytoplasm	enzyme	
−28.54	5.52E-03	ENSG00000116183		PAPPA2	pappalysin 2	Extracellular Space	peptidase	
−27.92	5.54E-03	ENSG00000135903		PAX3	paired box 3	Nucleus	transcription regulator	
27.72	5.54E-03	ENSG00000112852		PCDHB2	protocadherin beta 2	Plasma Membrane	other	
27.88	5.54E-03	ENSG00000254544		PCNAP4	proliferating cell nuclear antigen pseudogene 4	Other	other	
−17.15	2.07E-02	ENSG00000244302		PEX5L-AS2	PEX5L antisense RNA 2	Other	other	
−27.30	6.04E-03	ENSG00000233328		PFN1P1	profilin 1 pseudogene 1	Other	other	
−26.19	7.91E-03	ENSG00000124102		PI3	peptidase inhibitor 3	Extracellular Space	other	
−19.50	4.63E-03	ENSG00000007541		PIGQ	phosphatidylinositol glycan anchor biosynthesis class Q	Cytoplasm	enzyme	
−18.52	7.91E-02	ENSG00000135549		PKIB	cAMP-dependent protein kinase inhibitor beta	Other	other	
−28.77	5.52E-03	ENSG00000170890		PLA2G1B	phospholipase A2 group IB	Extracellular Space	enzyme	niflumic acid
−29.77	4.30E-03	ENSG00000104356		POP1	POP1 homolog, ribonuclease P/MRP subunit	Nucleus	enzyme	
−21.53	7.44E-03	ENSG00000271776		POU4F1	POU class 4 homeobox 1	Nucleus	transcription regulator	
−25.71	9.87E-03	ENSG00000233319		PPIAP32	peptidylprolyl isomerase A pseudogene 32	Other	other	
18.01	4.35E-02	ENSG00000068971		PPP2R5B	protein phosphatase 2 regulatory subunit B'beta	Cytoplasm	phosphatase	
26.50	7.91E-03	ENSG00000126583		PRKCG	protein kinase C gamma	Cytoplasm	kinase	ingenol mebutate
−30.00	4.30E-03	ENSG00000237943		PRKCQ-AS1	PRKCQ antisense RNA 1	Other	other	
−30.00	4.30E-03	ENSG00000255251	D	PRR23D1/PRR23D2	proline rich 23 domain containing 1	Other	other	
−29.92	4.30E-03	ENSG00000255378	D	PRR23D1/PRR23D2	proline rich 23 domain containing 1	Other	other	
−19.23	7.91E-03	ENSG00000080815		PSEN1	presenilin 1	Plasma Membrane	peptidase	tarenflurbil
−26.19	7.91E-03	ENSG00000240065		PSMB9	proteasome subunit beta 9	Cytoplasm	peptidase	carfilzomib, carfilzomib/dexamethasone/lenalidomide, carfilzomib/dexamethasone/rituximab, carfilzomib/dexamethasone
−28.52	5.52E-03	ENSG00000227462		PSME2P6	proteasome activator subunit 2 pseudogene 6	Other	other	
−27.92	5.54E-03	ENSG00000244694		PTCHD4	patched domain containing 4	Other	other	
−26.36	7.91E-03	ENSG00000171611		PTCRA	pre T-cell antigen receptor alpha	Plasma Membrane	other	
26.50	7.91E-03	ENSG00000172733		PURG	purine rich element binding protein G	Other	other	
30.00	4.30E-03	ENSG00000155961		RAB39B	RAB39B, member RAS oncogene family	Plasma Membrane	enzyme	
−26.19	7.91E-03	ENSG00000198774		RASSF9	Ras association domain family member 9	Cytoplasm	transporter	
−30.00	4.30E-03	ENSG00000133119		RFC3	replication factor C subunit 3	Nucleus	enzyme	
27.58	5.67E-03	ENSG00000168411		RFWD3	ring finger and WD repeat domain 3	Nucleus	enzyme	
−27.97	5.54E-03	ENSG00000102760		RGCC	regulator of cell cycle	Cytoplasm	other	
−29.62	4.37E-03	ENSG00000101883		RHOXF1	Rhox homeobox family member 1	Nucleus	other	
28.01	5.54E-03	ENSG00000234493		RHOXF1P1	Rhox homeobox family member 1 pseudogene 1	Other	other	
−28.49	5.52E-03	ENSG00000252490		RN7SKP66		Other	other	
27.91	5.54E-03	ENSG00000199940		RN7SKP75		Other	other	
−27.92	5.54E-03	ENSG00000200243		RN7SKP79	RNA, 7SK small nuclear pseudogene 79	Other	other	
−28.12	5.54E-03	ENSG00000266863		RN7SL123P	RNA, 7SL, cytoplasmic 123, pseudogene	Other	other	
−29.55	4.37E-03	ENSG00000239279		RN7SL184P	RNA, 7SL, cytoplasmic 184, pseudogene	Other	other	
30.00	4.30E-03	ENSG00000265740		RN7SL339P		Other	other	
28.09	5.54E-03	ENSG00000240577		RN7SL445P	RNA, 7SL, cytoplasmic 445, pseudogene	Other	other	
−28.80	5.52E-03	ENSG00000239504		RN7SL583P	RNA, 7SL, cytoplasmic 583, pseudogene	Other	other	
26.85	7.20E-03	ENSG00000244264		RN7SL597P		Other	other	
−29.14	4.77E-03	ENSG00000239744		RN7SL63P		Other	other	
30.00	4.30E-03	ENSG00000239319		RN7SL854P		Other	other	
−29.77	4.30E-03	ENSG00000202263		RNA5SP22	RNA, 5S ribosomal pseudogene 22	Other	other	
−26.19	7.91E-03	ENSG00000202225		RNA5SP240	RNA, 5S ribosomal pseudogene 240	Other	other	
−27.92	5.54E-03	ENSG00000199354		RNA5SP273	RNA, 5S ribosomal pseudogene 273	Other	other	
26.83	7.14E-03	ENSG00000104889		RNASEH2A	ribonuclease H2 subunit A	Nucleus	enzyme	
−26.36	7.91E-03	ENSG00000250896		RNPS1P1	RNA binding protein with serine rich domain 1 pseudogene 1	Other	other	
−28.49	5.52E-03	ENSG00000202313		RNU1-64P		Other	other	
−25.48	1.15E-02	ENSG00000252018		RNU2-30P	RNA, U2 small nuclear 30, pseudogene	Other	other	
−26.36	7.91E-03	ENSG00000253081		RNU4ATAC17P	RNA, U4atac small nuclear 17, pseudogene	Other	other	
−28.55	5.52E-03	ENSG00000206931		RNU6-1042P	RNA, U6 small nuclear 1042, pseudogene	Other	other	
−27.92	5.54E-03	ENSG00000201165		RNU6-1229P	RNA, U6 small nuclear 1229, pseudogene	Other	other	
−28.03	5.54E-03	ENSG00000207312		RNU6-429P	RNA, U6 small nuclear 429, pseudogene	Other	other	
30.00	4.30E-03	ENSG00000206983		RNU6-49P	RNA, U6 small nuclear 49, pseudogene	Other	other	
−25.48	1.15E-02	ENSG00000252860		RNU6-570P	RNA, U6 small nuclear 570, pseudogene	Other	other	
−27.42	5.67E-03	ENSG00000222972		RNU6-651P		Other	other	
−27.02	7.01E-03	ENSG00000207333		RNU6-680P		Other	other	
26.50	7.91E-03	ENSG00000207058		RNU6-784P		Other	other	
−28.49	5.52E-03	ENSG00000206960		RNU6-793P	RNA, U6 small nuclear 793, pseudogene	Other	other	
28.50	5.52E-03	ENSG00000238788		RNU7-182P	RNA, U7 small nuclear 182 pseudogene	Other	other	
−26.19	7.91E-03	ENSG00000104237		RP1	RP1, axonemal microtubule associated	Cytoplasm	other	
−27.92	5.54E-03	ENSG00000230853		RPL10P11	ribosomal protein L10 pseudogene 11	Other	other	
26.47	7.91E-03	ENSG00000240370		RPL13P5	ribosomal protein L13 pseudogene 5	Other	other	
−29.92	4.30E-03	ENSG00000230559		RPL17P12	ribosomal protein L17 pseudogene 12	Other	other	
−26.96	7.09E-03	ENSG00000243964		RPL23AP65	ribosomal protein L23a pseudogene 65	Other	other	
30.00	4.30E-03	ENSG00000230364		RPL4P3	ribosomal protein L4 pseudogene 3	Other	other	
28.23	5.54E-03	ENSG00000243914		RPL5P14	ribosomal protein L5 pseudogene 14	Other	other	
−28.29	5.52E-03	ENSG00000243521		RPL5P33	ribosomal protein L5 pseudogene 33	Other	other	
−28.17	5.54E-03	ENSG00000234415		RPL5P7	ribosomal protein L5 pseudogene 7	Other	other	
−26.19	7.91E-03	ENSG00000243742		RPLP0P2	ribosomal protein lateral stalk subunit P0 pseudogene 2	Other	other	
−27.92	5.54E-03	ENSG00000163684		RPP14	ribonuclease P/MRP subunit p14	Nucleus	enzyme	
26.13	8.21E-03	ENSG00000180172		RPS12P23	ribosomal protein S12 pseudogene 23	Other	other	
−27.30	6.04E-03	ENSG00000236862		RPS20P24	ribosomal protein S20 pseudogene 24	Other	other	
−29.50	4.37E-03	ENSG00000231241		RPS3AP3	ribosomal protein S3A pseudogene 3	Other	other	
26.47	7.91E-03	ENSG00000226525		RPS7P10	ribosomal protein S7 pseudogene 10	Cytoplasm	other	
29.99	4.30E-03	ENSG00000228820		RPSAP1	ribosomal protein SA pseudogene 1	Other	other	
−26.96	7.09E-03	ENSG00000227721		RPSAP64	ribosomal protein SA pseudogene 64	Other	other	
26.83	7.14E-03	ENSG00000235598		RRM2P4	ribonucleotide reductase M2 polypeptide pseudogene 4	Other	other	
30.00	4.30E-03	ENSG00000166562		SEC11C	SEC11 homolog C, signal peptidase complex subunit	Cytoplasm	peptidase	
−28.50	5.52E-03	ENSG00000138623		SEMA7A	semaphorin 7A (John Milton Hagen blood group)	Plasma Membrane	transmembrane receptor	
−30.00	4.30E-03	ENSG00000240489		SETP14	SET pseudogene 14	Other	other	
−26.19	7.91E-03	ENSG00000090402		SI	sucrase-isomaltase	Cytoplasm	enzyme	
27.73	5.66E-03	ENSG00000225774		SIRPAP1	signal regulatory protein alpha pseudogene 1	Other	other	
26.47	7.91E-03	ENSG00000179520		SLC17A8	solute carrier family 17 member 8	Plasma Membrane	transporter	
−26.69	7.65E-03	ENSG00000105143		SLC1A6	solute carrier family 1 member 6	Plasma Membrane	transporter	riluzole
−27.02	7.01E-03	ENSG00000183048		SLC25A10	solute carrier family 25 member 10	Cytoplasm	transporter	
−18.95	3.62E-02	ENSG00000225347		SLC25A5P8	solute carrier family 25 member 5 pseudogene 8	Other	other	
−27.92	5.54E-03	ENSG00000251078		SLC25A5P9	solute carrier family 25 member 5 pseudogene 9	Other	other	
30.00	4.30E-03	ENSG00000124786		SLC35B3	solute carrier family 35 member B3	Cytoplasm	transporter	
−27.02	7.01E-03	ENSG00000188338		SLC38A3	solute carrier family 38 member 3	Plasma Membrane	transporter	
−27.71	5.66E-03	ENSG00000148482		SLC39A12	solute carrier family 39 member 12	Plasma Membrane	transporter	
−28.07	5.54E-03	ENSG00000214329		SLC9B1P2	solute carrier family 9 member B1 pseudogene 2	Other	other	
−26.19	7.91E-03	ENSG00000185985		SLITRK2	SLIT and NTRK like family member 2	Plasma Membrane	other	
28.14	5.52E-03	ENSG00000200354		SNORA71D	small nucleolar RNA, H/ACA box 71D	Other	other	
26.50	7.91E-03	ENSG00000201634		SNORD115-48	small nucleolar RNA, C/D box 115-48	Other	other	
26.50	7.91E-03	ENSG00000239043		SNORD127	small nucleolar RNA, C/D box 127	Other	other	
27.58	5.67E-03	ENSG00000201330		SNORD32B	small nucleolar RNA, C/D box 32B	Other	other	
28.72	5.52E-03	ENSG00000207215		SNORD3H	small nucleolar RNA, C/D box 3H	Other	other	
−28.49	5.52E-03	ENSG00000225642		SNRPEP5	small nuclear ribonucleoprotein polypeptide E pseudogene 5	Other	other	
−27.02	7.01E-03	ENSG00000228741		SPATA13	spermatogenesis associated 13	Plasma Membrane	other	
27.58	5.67E-03	ENSG00000258916		SPATA31E2P	SPATA31 subfamily E member 2, pseudogene	Other	other	
27.58	5.67E-03	ENSG00000274279		SPATA31E3P	SPATA31 subfamily E member 3, pseudogene	Other	other	
27.05	7.01E-03	ENSG00000164299		SPZ1	spermatogenic leucine zipper 1	Nucleus	transcription regulator	
−23.05	9.68E-03	ENSG00000138378		STAT4	signal transducer and activator of transcription 4	Nucleus	transcription regulator	
30.00	4.30E-03	ENSG00000165730		STOX1	storkhead box 1	Cytoplasm	other	
29.88	4.30E-03	ENSG00000164744		SUN3	Sad1 and UNC84 domain containing 3	Nucleus	other	
29.35	4.45E-03	ENSG00000217442		SYCE3	synaptonemal complex central element protein 3	Nucleus	other	
27.01	6.99E-03	ENSG00000127362		TAS2R3	taste 2 receptor member 3	Plasma Membrane	G-protein coupled receptor	
−28.49	5.52E-03	ENSG00000273513	D	TBC1D3 (includes others)	TBC1 domain family member 3	Extracellular Space	other	
20.61	9.19E-02	ENSG00000274611	D	TBC1D3 (includes others)	TBC1 domain family member 3	Extracellular Space	other	
−25.63	1.10E-02	ENSG00000236567		TCF3P1	transcription factor 3 pseudogene 1	Other	other	
−26.69	7.65E-03	ENSG00000167014		TERB2	telomere repeat binding bouquet formation protein 2	Nucleus	other	
−26.19	7.91E-03	ENSG00000135426		TESPA1	thymocyte expressed, positive selection associated 1	Cytoplasm	other	
26.85	7.20E-03	ENSG00000237675		TEX36-AS1	TEX36 antisense RNA 1	Other	other	
20.64	8.58E-02	ENSG00000226674		TEX41	testis expressed 41 (non-protein coding)	Other	other	
27.58	5.67E-03	ENSG00000008197		TFAP2D	transcription factor AP-2 delta	Nucleus	transcription regulator	
−26.19	7.91E-03	ENSG00000243926		TIPARP-AS1	TIPARP antisense RNA 1	Other	other	
−26.19	7.91E-03	ENSG00000151952		TMEM132D	transmembrane protein 132D	Other	other	
27.72	5.66E-03	ENSG00000178821		TMEM52	transmembrane protein 52	Other	other	
−28.87	5.42E-03	ENSG00000154646		TMPRSS15	transmembrane protease, serine 15	Extracellular Space	peptidase	
−26.36	7.91E-03	ENSG00000176040		TMPRSS7	transmembrane protease, serine 7	Plasma Membrane	peptidase	
−30.00	4.30E-03	ENSG00000235211		TMSB10P2	thymosin beta 10 pseudogene 2	Other	other	
−28.07	5.54E-03	ENSG00000188765		TMSB4XP2	thymosin beta 4, X-linked pseudogene 2	Other	other	
−30.00	4.30E-03	ENSG00000223551		TMSB4XP4	thymosin beta 4, X-linked pseudogene 4	Other	other	
−27.06	6.99E-03	ENSG00000248697		TOX4P1	TOX high mobility group box family member 4 pseudogene 1	Other	other	
25.98	8.69E-03	ENSG00000139287		TPH2	tryptophan hydroxylase 2	Plasma Membrane	enzyme	
−29.51	4.30E-03	ENSG00000234782		TPT1P9	tumor protein, translationally-controlled 1 pseudogene 9	Other	other	
26.50	7.91E-03	ENSG00000211831		TRAJ61	T cell receptor alpha joining 61 (non-functional)	Other	other	
26.13	8.21E-03	ENSG00000211880		TRAJ9	T cell receptor alpha joining 9	Other	other	
27.05	7.01E-03	ENSG00000211818		TRAV39	T cell receptor alpha variable 39	Other	other	
−27.42	6.04E-03	ENSG00000211710		TRBV4-1	T cell receptor beta variable 4-1	Other	other	
−30.00	4.30E-03	ENSG00000227550		TRBV7-5	T cell receptor beta variable 7–5 (pseudogene)	Other	other	
−29.70	4.30E-03	ENSG00000132109		TRIM21	tripartite motif containing 21	Nucleus	enzyme	
28.15	5.54E-03	ENSG00000174173		TRMT10C	tRNA methyltransferase 10C, mitochondrial RNase P subunit	Cytoplasm	enzyme	
−28.80	5.52E-03	ENSG00000134198		TSPAN2	tetraspanin 2	Extracellular Space	other	
−28.54	5.52E-03	ENSG00000197763		TXNRD3	thioredoxin reductase 3	Cytoplasm	enzyme	
−26.69	7.65E-03	ENSG00000215218		UBE2QL1	ubiquitin conjugating enzyme E2 Q family like 1	Nucleus	enzyme	
28.19	5.52E-03	ENSG00000229667		UBE2V1P9	ubiquitin conjugating enzyme E2 V1 pseudogene 9	Other	other	
−30.00	4.30E-03	ENSG00000165623		UCMA	upper zone of growth plate and cartilage matrix associated	Extracellular Space	other	
−28.07	5.54E-03	ENSG00000260722		VN1R67P	vomeronasal 1 receptor 67 pseudogene	Other	other	
−26.72	7.88E-03	ENSG00000251900		VTRNA2-2P		Other	other	
27.36	6.18E-03	ENSG00000229932		YWHAZP3	tyrosine 3-monooxygenase/tryptophan 5-monooxygenase activation protein zeta pseudogene 3	Other	other	
−28.07	5.54E-03	ENSG00000155329		ZCCHC10	zinc finger CCHC-type containing 10	Other	other	
27.86	5.54E-03	ENSG00000155256		ZFYVE27	zinc finger FYVE-type containing 27	Plasma Membrane	other	
28.23	5.52E-03	ENSG00000170631		ZNF16	zinc finger protein 16	Nucleus	other	
28.23	5.54E-03	ENSG00000204789		ZNF204P	zinc finger protein 204, pseudogene	Other	other	
−26.19	7.91E-03	ENSG00000185947		ZNF267	zinc finger protein 267	Nucleus	other	
−27.92	5.54E-03	ENSG00000186026		ZNF284	zinc finger protein 284	Other	other	
26.83	7.14E-03	ENSG00000180855		ZNF443	zinc finger protein 443	Nucleus	transcription regulator	
25.98	8.69E-03	ENSG00000188868		ZNF563	zinc finger protein 563	Nucleus	other	
−30.00	4.30E-03	ENSG00000198182		ZNF607	zinc finger protein 607	Nucleus	other	
−28.80	5.52E-03	ENSG00000018607		ZNF806	zinc finger protein 806	Other	other	
−28.07	5.54E-03	ENSG00000197933		ZNF823	zinc finger protein 823	Nucleus	other	+A3:I459

The data in Table 2 contains all the loci which did not map to a gene but were identified as significantly altered in the breast tumor by consumption of walnut using the same calculations as for the data in Table 1. The meaning of changes in these loci has not been identified.

**Table 2:** All unmapped loci. ^©^ 2000–2018 QIAGEN. All rights reserved.

Expr log ratio	Expr false discovery rate (q-value)	ID
−30	4.30E-03	ENSG00000206856
−30	4.30E-03	ENSG00000222133
−30	9.14E-04	ENSG00000248511
30	4.30E-03	ENSG00000249023
−30	4.30E-03	ENSG00000249239
−30	4.30E-03	ENSG00000249411
30	4.30E-03	ENSG00000249842
30	4.30E-03	ENSG00000250516
−30	4.30E-03	ENSG00000250908
30	4.30E-03	ENSG00000251066
−30	4.30E-03	ENSG00000254558
−30	4.30E-03	ENSG00000256306
−30	4.30E-03	ENSG00000259057
30	4.30E-03	ENSG00000260066
30	4.30E-03	ENSG00000264486
−30	4.30E-03	ENSG00000265621
30	4.30E-03	ENSG00000269524
30	4.30E-03	ENSG00000272108
−30	4.30E-03	ENSG00000277269
30	4.30E-03	ENSG00000279162
30	4.30E-03	ENSG00000279349
−30	4.30E-03	ENSG00000279957
30	4.30E-03	ENSG00000280068
−30	4.30E-03	ENSG00000281116
30	4.30E-03	ENSG00000254348
−30	4.30E-03	ENSG00000278777
−29.999	4.30E-03	ENSG00000251244
−29.988	4.30E-03	ENSG00000274918
−29.973	4.30E-03	ENSG00000273920
−29.947	4.30E-03	ENSG00000255305
−29.896	4.30E-03	ENSG00000261334
−29.878	4.30E-03	ENSG00000254242
−29.86	4.30E-03	ENSG00000278126
−29.818	4.30E-03	ENSG00000232306
−29.801	4.30E-03	ENSG00000271627
29.732	4.30E-03	ENSG00000221210
−29.718	4.30E-03	ENSG00000249818
−29.634	4.45E-03	ENSG00000220349
−29.58	4.37E-03	ENSG00000260317
29.547	4.30E-03	ENSG00000249189
−29.495	4.37E-03	ENSG00000257654
−29.495	4.37E-03	ENSG00000275228
29.495	4.37E-03	ENSG00000228935
−29.474	4.32E-03	ENSG00000263713
−29.465	4.37E-03	ENSG00000257863
−29.437	4.45E-03	ENSG00000232328
−29.424	4.45E-03	ENSG00000251448
29.4	4.37E-03	ENSG00000277598
29.354	4.45E-03	ENSG00000261835
−29.347	4.45E-03	ENSG00000272940
−29.287	4.62E-03	ENSG00000267286
29.239	4.62E-03	ENSG00000228438
29.172	4.70E-03	ENSG00000223738
−29.143	4.77E-03	ENSG00000231916
−29.143	4.77E-03	ENSG00000233703
29.134	4.77E-03	ENSG00000278192
29.068	4.71E-03	ENSG00000264642
−28.843	5.52E-03	ENSG00000232887
28.841	5.08E-03	ENSG00000249061
−28.802	5.52E-03	ENSG00000229370
−28.802	5.52E-03	ENSG00000233372
−28.802	5.52E-03	ENSG00000235147
−28.802	5.52E-03	ENSG00000258314
28.679	5.52E-03	ENSG00000274444
28.566	5.52E-03	ENSG00000264526
−28.548	5.52E-03	ENSG00000256293
−28.539	5.52E-03	ENSG00000229242
−28.539	5.52E-03	ENSG00000240103
−28.539	5.52E-03	ENSG00000257129
−28.539	5.52E-03	ENSG00000269514
−28.518	5.52E-03	ENSG00000276538
−28.517	5.52E-03	ENSG00000281411
−28.492	5.52E-03	ENSG00000232140
−28.492	5.52E-03	ENSG00000234941
−28.492	5.52E-03	ENSG00000237903
−28.492	5.52E-03	ENSG00000248656
−28.492	5.52E-03	ENSG00000260093
−28.492	5.52E-03	ENSG00000274370
−28.311	5.52E-03	ENSG00000273816
−28.29	5.52E-03	ENSG00000281114
28.23	5.54E-03	ENSG00000202269
28.23	5.54E-03	ENSG00000232028
28.23	5.54E-03	ENSG00000239288
28.23	5.54E-03	ENSG00000261679
28.23	5.54E-03	ENSG00000270866
28.188	5.52E-03	ENSG00000232120
28.188	5.52E-03	ENSG00000234683
28.188	5.52E-03	ENSG00000282950
28.154	5.54E-03	ENSG00000255851
28.154	5.54E-03	ENSG00000281046
28.144	5.52E-03	ENSG00000250847
28.144	5.52E-03	ENSG00000261680
28.094	5.54E-03	ENSG00000233616
−28.083	5.52E-03	ENSG00000278923
28.077	5.54E-03	ENSG00000244167
−28.068	5.54E-03	ENSG00000249102
−28.068	5.54E-03	ENSG00000263717
−28.068	5.54E-03	ENSG00000264182
28.006	5.54E-03	ENSG00000251668
27.985	5.54E-03	ENSG00000256029
27.985	5.54E-03	ENSG00000280164
−27.971	5.54E-03	ENSG00000249631
−27.924	5.54E-03	ENSG00000225148
−27.924	5.54E-03	ENSG00000255572
−27.924	5.54E-03	ENSG00000260329
−27.924	5.54E-03	ENSG00000270889
−27.924	5.54E-03	ENSG00000272685
27.914	5.54E-03	ENSG00000279127
27.862	5.54E-03	ENSG00000283057
−27.829	5.54E-03	ENSG00000271568
−27.779	5.54E-03	ENSG00000254502
−27.779	5.54E-03	ENSG00000263985
−27.747	5.54E-03	ENSG00000254290
27.733	5.66E-03	ENSG00000227133
27.733	5.66E-03	ENSG00000243831
27.733	5.66E-03	ENSG00000259924
27.733	5.66E-03	ENSG00000273259
27.733	5.66E-03	ENSG00000279708
27.722	5.66E-03	ENSG00000241961
27.719	5.54E-03	ENSG00000258205
−27.71	5.66E-03	ENSG00000260674
27.644	5.67E-03	ENSG00000254792
27.644	5.67E-03	ENSG00000225720
27.579	5.67E-03	ENSG00000260261
27.579	5.67E-03	ENSG00000265819
27.579	5.67E-03	ENSG00000272512
27.579	5.67E-03	ENSG00000279485
27.579	5.67E-03	ENSG00000280023
27.579	5.67E-03	ENSG00000280188
−27.496	5.66E-03	ENSG00000199285
27.477	5.67E-03	ENSG00000238009
27.477	5.67E-03	ENSG00000241168
27.477	5.67E-03	ENSG00000258271
27.477	5.67E-03	ENSG00000259450
27.477	5.67E-03	ENSG00000261379
−27.424	6.04E-03	ENSG00000282173
27.407	6.04E-03	ENSG00000201535
27.357	6.18E-03	ENSG00000278269
−27.323	6.26E-03	ENSG00000263938
−27.058	6.99E-03	ENSG00000269981
−27.058	6.99E-03	ENSG00000274815
27.051	7.01E-03	ENSG00000279122
−27.022	7.01E-03	ENSG00000217512
−27.022	7.01E-03	ENSG00000221376
−27.022	7.01E-03	ENSG00000264855
−27.022	7.01E-03	ENSG00000269974
−27.022	7.01E-03	ENSG00000271118
−27.018	7.01E-03	ENSG00000224661
−27.018	7.01E-03	ENSG00000233645
−27.018	7.01E-03	ENSG00000234622
−27.018	7.01E-03	ENSG00000244259
−27.018	7.01E-03	ENSG00000264715
−27.018	7.01E-03	ENSG00000265445
−27.018	7.01E-03	ENSG00000267004
−27.018	7.01E-03	ENSG00000271253
−27.018	7.01E-03	ENSG00000275958
27.005	6.99E-03	ENSG00000225591
27.005	6.99E-03	ENSG00000229791
−26.96	7.09E-03	ENSG00000207027
−26.96	7.09E-03	ENSG00000229878
−26.96	7.09E-03	ENSG00000233778
−26.96	7.09E-03	ENSG00000261620
−26.96	7.09E-03	ENSG00000264222
−26.91	7.01E-03	ENSG00000256783
26.854	7.20E-03	ENSG00000277930
26.854	7.20E-03	ENSG00000279173
26.826	7.14E-03	ENSG00000201370
26.826	7.14E-03	ENSG00000202410
26.826	7.14E-03	ENSG00000224904
26.826	7.14E-03	ENSG00000240159
26.761	7.20E-03	ENSG00000230687
26.761	7.20E-03	ENSG00000258773
26.761	7.20E-03	ENSG00000260702
−26.688	7.65E-03	ENSG00000224945
−26.688	7.65E-03	ENSG00000226986
−26.688	7.65E-03	ENSG00000271993
−26.688	7.65E-03	ENSG00000280395
−26.688	7.65E-03	ENSG00000281304
26.498	7.91E-03	ENSG00000236209
26.498	7.91E-03	ENSG00000237555
26.469	7.91E-03	ENSG00000213184
26.469	7.91E-03	ENSG00000231297
26.469	7.91E-03	ENSG00000233428
26.469	7.91E-03	ENSG00000253816
26.469	7.91E-03	ENSG00000262089
26.469	7.91E-03	ENSG00000267284
26.469	7.91E-03	ENSG00000273100
26.469	7.91E-03	ENSG00000274964
26.469	7.91E-03	ENSG00000277169
26.469	7.91E-03	ENSG00000277875
26.469	7.91E-03	ENSG00000279537
26.469	7.91E-03	ENSG00000280198
−26.362	7.91E-03	ENSG00000199901
−26.362	7.91E-03	ENSG00000206672
−26.362	7.91E-03	ENSG00000206806
−26.362	7.91E-03	ENSG00000213279
−26.362	7.91E-03	ENSG00000254367
−26.362	7.91E-03	ENSG00000259275
−26.362	7.91E-03	ENSG00000262766
−26.362	7.91E-03	ENSG00000265359
−26.362	7.91E-03	ENSG00000267717
−26.362	7.91E-03	ENSG00000269014
−26.362	7.91E-03	ENSG00000271897
−26.362	7.91E-03	ENSG00000271917
−26.362	7.91E-03	ENSG00000277026
−26.362	7.91E-03	ENSG00000279269
−26.362	7.91E-03	ENSG00000279910
−26.362	7.91E-03	ENSG00000280114
−26.362	7.91E-03	ENSG00000280711
−26.299	7.91E-03	ENSG00000236116
−26.192	7.91E-03	ENSG00000204556
−26.192	7.91E-03	ENSG00000207229
−26.192	7.91E-03	ENSG00000221326
−26.192	7.91E-03	ENSG00000227740
−26.192	7.91E-03	ENSG00000228176
−26.192	7.91E-03	ENSG00000228543
−26.192	7.91E-03	ENSG00000233516
−26.192	7.91E-03	ENSG00000233887
−26.192	7.91E-03	ENSG00000235358
−26.192	7.91E-03	ENSG00000248490
−26.192	7.91E-03	ENSG00000249284
−26.192	7.91E-03	ENSG00000254330
−26.192	7.91E-03	ENSG00000254836
−26.192	7.91E-03	ENSG00000255344
−26.192	7.91E-03	ENSG00000260603
−26.192	7.91E-03	ENSG00000261618
−26.192	7.91E-03	ENSG00000265554
−26.192	7.91E-03	ENSG00000268597
−26.192	7.91E-03	ENSG00000271643
−26.192	7.91E-03	ENSG00000274421
−26.192	7.91E-03	ENSG00000276073
−26.192	7.91E-03	ENSG00000278909
−26.192	7.91E-03	ENSG00000280061
−26.192	7.91E-03	ENSG00000281262
26.132	8.21E-03	ENSG00000217120
26.132	8.21E-03	ENSG00000231705
26.132	8.21E-03	ENSG00000243779
26.132	8.21E-03	ENSG00000271364
26.132	8.21E-03	ENSG00000276507
−25.998	8.63E-03	ENSG00000265985
25.983	8.69E-03	ENSG00000223672
25.983	8.69E-03	ENSG00000225193
25.983	8.69E-03	ENSG00000254436
25.983	8.69E-03	ENSG00000256494
25.983	8.69E-03	ENSG00000261696
25.983	8.69E-03	ENSG00000263606
25.983	8.69E-03	ENSG00000267027
25.983	8.69E-03	ENSG00000273348
25.983	8.69E-03	ENSG00000280245
−25.71	9.87E-03	ENSG00000220076
−25.71	9.87E-03	ENSG00000253960
−25.71	9.87E-03	ENSG00000260244
−25.71	9.87E-03	ENSG00000279077
25.687	9.93E-03	ENSG00000199635
25.687	9.93E-03	ENSG00000276337
−25.633	1.10E-02	ENSG00000228035
−25.633	1.10E-02	ENSG00000239036
−25.633	1.10E-02	ENSG00000263489
−25.484	1.15E-02	ENSG00000242953
−25.484	1.15E-02	ENSG00000255774
−22.956	1.33E-02	ENSG00000274522
22.792	3.30E-02	ENSG00000270019
22.383	3.50E-02	ENSG00000272461
22.266	1.01E-02	ENSG00000278233
−21.352	7.91E-03	ENSG00000239381
−20.711	8.68E-02	ENSG00000276668
20.55	9.19E-02	ENSG00000278824
19.815	7.91E-03	ENSG00000243429
−19.642	7.91E-03	ENSG00000272366
−19.632	2.85E-02	ENSG00000255740
−19.491	5.52E-03	ENSG00000227777
18.824	4.75E-02	ENSG00000272763
−18.033	6.99E-03	ENSG00000275188
17.984	8.53E-02	ENSG00000256757
−17.983	1.03E-02	ENSG00000255871
−16.819	5.84E-02	ENSG00000265129
−16.575	9.30E-04	ENSG00000233875
16.457	5.88E-03	ENSG00000237158
−16.035	4.59E-02	ENSG00000268906
−15.501	5.14E-02	ENSG00000201892
−2.414	3.36E-02	ENSG00000277089

IPA analyses use the results shown in Table 1 to organize the genes into functional networks. This is important to identify the net effect of multiple changes on gene expression. The data in Table 3 lists all 25 significantly modified gene networks that were identified by IPA analyses, the genes in those networks and the top diseases and functions associated with the genes. The network ‘score’, the negative log of the overall statistical significance of the network, is shown. A network score of 41 means that in 10^−41^ experiments of a similar type one might to encounter this pattern of mRNA expression changes by chance. The data in this table indicates the effect of walnut consumption on gene networks in the existing breast cancer and could indicate other diseases or functions in which dietary walnut may have benefit. Focused research would be needed to ascertain this benefit.

**Table 3:** All gene networks identified by IPA analyses and the molecules in that network. © 2000–2018 QIAGEN. All rights reserved.

ID	Molecules in Network	Score	Focus Molecules	Top Diseases and Functions
1	ADGRG4,AJAP1,AVPR1B,BDKRB1,CD3,CDH9,DAB1,DDX4,DPYSL5,ECH1,ERK,estrogen receptor,FAM49A,Gpcr,Histone h3,HOXB5,Insulin,Jnk,MRPS9,NMUR1,NTSR1,OPRL1,P38 MAPK,PAPPA2,PI3K (complex),Pkc(s),PLC,POU4F1,PSEN1,SI,SLC38A3,TCR,TFAP2D,TSPAN2,ZCCHC10	41	23	Reproductive System Development and Function, Carbohydrate Metabolism, Lipid Metabolism
3	ADCY,Akt,AMPK,BMP2,CCL14,CDH18,Cg,CHRM2,Collagen(s),Creb,DEFA6,GFRA4,Growth hormone,GZMA,HTR7,KCNJ3,Mapk,MAPK4,mir-15,NDP,NOX4,NR2E1,PDGF BB,Pka,PPP2R5B,Proinsulin,Ras,SEMA7A,Serine Protease,SLC39A12,TMPRSS7,TMPRSS15,Tnf (family),trypsin, Vegf	32	19	Embryonic Development, Nervous System Development and Function, Organ Development
4	C1q,CD1A,CLEC4A,CLEC4M,CR2,CSN2,CYP46A1,ERK1/2,FCRLA,GFRA2,IAPP,IFI44,IFN alpha/beta,IFN Beta,Ifnar,IgG,IgG1,IgG2a,IgG2b,Igm,IL1,IL23,IL12 (complex),IL23R,Immunoglobulin,Interferon alpha,LDL,PI3,Pro-inflammatory Cytokine,PSMB9,RNASEH2A,SPZ1,STAT4,Tgf beta,TRIM21	27	17	Endocrine System Disorders, Gastrointestinal Disease, Immunological Disease
5	ADAT1,AGPAT1,ARHGEF4,CCDC134,CD200,CELF5,ELAVL2,GAD,H2AFB3 (includes others),HIST1H2BO,IFI44,IFNG,IGFN1,IL5,IL6,KLK8,LCP2,LECT2,MAPK8,MDGA2,MOV10,MRPL22,NRTN,OSBP2,PAIP2B,PI3,PIGQ,PKIB,RP1,SIRPB1,SLC35B3,SUN3,TESPA1,TNFSF13B,ZNF443	25	16	Cell-To-Cell Signaling and Interaction, Cellular Growth and Proliferation, Hematological System Development and Function
6	ACTA2,Actn3,AUH,C11orf86,C3orf38,C3orf79,CHST12,COL27A1,FN1,HEATR5B,IL4,KLHL1,MARCKS,Mcpt4,MUC17,Mucin,NDST2,OPRPN,OXLD1,PI3,PPARD,PSMG1,RAB10,RAB33B,RAB39B,RGCC,SEC11C,SLC1A6,SPEF2,SPG11,STK32C,STOX1,SYT2,TRAPPC6B,ZFYVE27	23	15	Cell-To-Cell Signaling and Interaction, Carbohydrate Metabolism, Drug Metabolism
7	APP,BNIP1,C18orf21,CALML3,CBFA2T2,CCDC140,CLEC12A,DTD1,FOLR1,GOSR2,GTF2E1,HIST1H2BM,KCNIP4,KCNIP4-IT1,KCNN3,MESDC2,NEPRO,PCBD2,POP1,POP4,POP5,POP7,PURG,RAD51C,RHOXF1,RPP14,RPP21,RPP25,RPP30,RPP40,RSPH10B/RSPH10B2,SURF2,TBC1D10C,TERB2,VTN	21	14	RNA Post-Transcriptional Modification, Cell-To-Cell Signaling and Interaction, Skeletal and Muscular System Development and Function
8	ABHD10,ABRAXAS1,ABRAXAS2,Adaptor protein 2,ADCYAP1,ANKRD40,ATP2C1,BDNF,BRCA1,BUD13,CCBE1,CCNI,CHSY1,CT45A10/CT45A5,DUSP27,ELAVL1,FAM46C,FLYWCH1,GOLT1B,HCN1,HCN3,HOXC12,LMBRD1,MLEC,MYADM,NABP1,NABP2,PPTC7,RPRM,SDCBP,SELENOT,SLC17A8,TBC1D3 (includes others),USP7,YPEL5	19	13	Amino Acid Metabolism, Molecular Transport, Small Molecule Biochemistry
9	ABT1,ANKRD30BP2,CCDC153,CNBP,COX10,CST5,EMP3,ESPNL,FKTN,FOLH1,GAR1,HGD,MED11,MED18,MED30,MED12L,MED13L,NAV3,NDUFB7,NIP7,NOP53,NR3C1,OLAH,PAK1,PARP4,PPARG,PPIC,PPP1R14C,SMARCA4,SPEN,SURF6,TRAP/Media,Wap,ZNF16,ZNF823	19	13	Cellular Assembly and Organization, Cellular Compromise, Gastrointestinal Disease
10	AK7,AQP3,AQP12A/AQP12B,ARNT2,BLOC1S3,BLOC1S4,BLOC1S5,BMPER,CCDC134,CDH3,COL12A1,COMP,DAND5,ESR2,GALNT4,GGCT,KCMF1,MAPK4,MTX3,NR5A2,PAFAH2,POLD4,RASSF7,RASSF9,RBP3,SAPCD2,SNRK,SOX2,SOX13,sPla2,TNF,TXNRD3,XPO1,ZFP36L2,ZNF267	17	12	Cancer, Organismal Injury and Abnormalities, Reproductive System Disease
11	ANXA3,AR,ATP13A4,BOK,CFAP206,CLDN3,CUL2,CYLC2,DDT,EDN2,ERBIN,GGCT,GUCY1A3,HLA-DQB2,KRT40,KTI12,LRRC1,MARCH4,Marcks,MIIP,MLKL,NUDT1,PDIA5,PFKFB2,PPP1CA,PRL,PTCHD4,SCN4A,SNW1,SYCE3,TMEM132D,TNFRSF10A,TP53,UCMA,ZNF563	17	12	Organ Morphology, Reproductive System Development and Function, Tissue Morphology
12	ADAM9,ADAM29,ADAM30,ADAMTS2,ADAMTS7,AHCTF1,CEACAM,CST4,CTNNB1,DACT2,DBH,DHDH,EGFR,FERD3L,FZD9,GRID1,IGLL1/IGLL5,IL2RA,IQGAP2,JADE1,LFNG,MDFI,Metalloprotease,MFNG,MYF5,PAPPA2,PTCRA,RAB18,SLC25A10,SPATA13,STX2,TCF3,TRMT10C,TSC22D1,ZNF607	15	11	Cell-mediated Immune Response, Lymphoid Tissue Structure and Development, Hematopoiesis
13	ACP5,ALPL,ARG1,CCNC,CXorf21,ELOA,EXOSC8,GADD45B,GTF2A1,GTF2A2,GTF2E2,GTF2F1,GTF2F2,GTF2H1,GTF2H3,HAS2,HMGN1,MED13,MSX2,MTPN,PCDHB2,POLR2G,POLR2J2/POLR2J3,RNA pol2-transcription factor,RNA polymerase II,S100A6,SBDS,SNAI2,SPP1,TCEA1,TCEA2,TFIIA,TFIIE,TFIIF,UBE2QL1	5	5	Gene Expression, Dental Disease, Digestive System Development and Function
14	FAM90A1,FAM90A26 (includes others)	2	1	Cancer, Neurological Disease, Organismal Injury and Abnormalities
15	CBLN2,miR-125b-5p (and other miRNAs w/seed CCCUGAG)	2	1	Cell Morphology, Cellular Function and Maintenance, Hematological System Development and Function
16	LHX2,OR8B8	2	1	Cellular Development, Cellular Growth and Proliferation, Digestive System Development and Function
17	mir-3146,miR-3146 (miRNAs w/seed AUGCUAG)	2	1	
18	mir-933,miR-933 (miRNAs w/seed GUGCGCA)	2	1	Cancer, Organismal Injury and Abnormalities, Reproductive System Disease
19	MIR4737,miR-4737 (miRNAs w/seed UGCGAGG)	2	1	
20	ANKRD30BL,HR	2	1	Dermatological Diseases and Conditions, Developmental Disorder, Embryonic Development
21	mir-3118,miR-3118 (and other miRNAs w/seed GUGACUG)	2	1	Cell Morphology, Cell-To-Cell Signaling and Interaction, Nervous System Development and Function
22	T2r,TAS2R3	2	1	Behavior, Cancer, Organismal Injury and Abnormalities
23	ZNF221,ZNF223,ZNF284	2	1	Cancer, Neurological Disease, Organismal Injury and Abnormalities
24	MIR6757,miR-6757-3p (miRNAs w/seed ACACUGG),miR-6757-5p (miRNAs w/seed AGGGAUG)	2	1	
25	NFYB,PTPRD,PTPRS,SLITRK2	2	1	Cellular Growth and Proliferation, Developmental Disorder, Endocrine System Disorders

## 2 Experimental design, materials and methods

### 2.1 Experimental design

Women were recruited for this clinical trial when they came to the clinic for their first diagnostic biopsy, before it was known if the lump was cancer or not. Potential subjects must have had lumps large enough for the necessary biopsies for diagnosis and 1 or 2 extra research biopsies. After signing informed consent, subjects were randomized into walnut-consuming or control groups. Subjects in the walnut group immediately began to consume 2 ounces of walnuts per day until surgery. If a subject was later found to not have cancer or if the cancer was to be treated with chemotherapy or radiation prior to surgery, she was no longer included in the trial. Thirty-eight women were initially recruited. Twenty-four of 38 subjects were disqualified because the lump was benign, or the subject was to receive chemo- or radiation therapy prior to surgery. An additional 4 subjects were disqualified because the extracted mRNA of at least one specimen did not pass quality control. Remaining were 10 subjects; five in the walnut consuming and five in the control group. mRNA was extracted from each individual specimen then genome wide mRNA was determined in each specimen via next-generation sequencing. Gene expression ratios were calculated for each gene as: (walnut surgery/walnut biopsy)/(control surgery/control biopsy) for further analyses [1].

### 2.2 IRB approval

The Marshall University Office of Research Integrity has an Institutional Review Board (IRB), which reviews and monitors all human subject research conducted at Marshall University, St. Mary's Medical Center, Cabell Huntington Hospital and the Edwards Cancer Center. The research protocol and participant informed consent were approved by the IRB (protocol number **339384–3)**. This study was not listed at ClinicalTrials.gov. Potential study participants were identified from records review by the Research Study Nurse prior to their appointment for a diagnostic biopsy. At the appointment time, the potential participant was interviewed by the study nurse, the study was explained and informed consent was obtained. The physician obtained one or two additional biopsies for research use when the biopsy was obtained for pathology studies.

**Inclusion criteria:** All subjects: 1) were female and with a breast mass that, according to standard of care, was to be biopsied for diagnosis and was large enough to obtain the needed biopsies for pathology and research; 2) understood and were willing to sign the informed consent form; 3) had an ECOG (Eastern Cooperative Oncology Group) performance status of 0 or 1; (0 - Fully active, able to carry on all pre-disease performance without restriction. 1 - Restricted in physically strenuous activity but ambulatory and able to carry out work of a light or sedentary nature, e.g., light housework, office work.) 4) were between 18 and 90 years of age; 5) were recruited as available without regard to race or ethnicity.

**Exclusion criteria:** Excluded persons were: 1) those who do not like or who were allergic to walnuts or other tree nuts; 2) those with any metabolic disease that could be affected by walnut consumption; 3) those with a life expectancy less than 6 months; 4) those who were pregnant (to prevent confounding due to pregnancy hormonal factors).

### 2.3 Clinical protocol

Subjects were consented at their first visit and were randomized into treated (consume walnut) or control (no added walnuts) groups. Routine clinical data were recorded (age, weight, height, family history, etc.). A five ml blood specimen in EDTA was collected for the research laboratory. After the initial biopsy, the subject was asked to continue to consume the usual diet and to not change consumption of any medication or supplements. If she was randomized to the walnut group, the subject was given 30, one ounce packages of walnuts, was asked to consume two packages (two ounces) of walnuts daily and to return remaining packages for counting. If needed, due to extended time for the clinical workup, the subject was given additional packages of walnuts to allow for continued consumption of two ounces of walnuts per day until surgery (about two to three weeks). Control group subjects were asked to not intentionally consume walnuts. At the conclusion of the study, each subject was asked to identify whether any changes were made to the usual diet especially in the areas of fruits, vegetables, nuts or supplement consumption and fats used in cooking and whether walnuts were consumed (walnut group) or not (control group).

At the time that ultrasound guided core needle biopsies of the breast mass were obtained for diagnosis, one or two extra cores were taken for research use. In the procedure room, immediately upon removal, the biopsies for research were placed in Qiagen All-Protect tissue reagent (Qiagen.com) to preserve RNA, DNA and protein for up to 7 days at room temperature. Biopsies were delivered to the research laboratory for initial processing within 2 hours.

If the pathology report indicated that the lump was not cancer, no further tissue was collected and the subject was no longer part of the study. If the biopsied tissue was breast cancer and surgery was scheduled without intervening radiation or chemotherapy, another specimen of tumor tissue and blood was collected at surgery. A small section of macroscopically viable tumor, away from the clean margin, was excised then immediately placed in Qiagen All-Protect tissue reagent, as before. Any patient who, according to the clinical care plan, was to receive either chemotherapy or radiation prior to surgery was no longer part of the study so as to not confound analyses.

### 2.4 Laboratory protocols

Total RNA was extracted using RNeasy, Lipid Tissue kit (Qiagen.com). This micro-kit is suitable for less than five mg of tissue and for extracting up to 45 μg of total mRNA. mRNA was checked for quantity and sent to the Marshall University Genomics Core Facility for further processing. The Genomics Core is a full service facility and provided RNA quality assessment, RNA-Seq analysis on each specimen, and DESeq2 expression profiling analyses of the data.

### 2.5 RNA sequencing: next-generation sequencing

RNA sample quality was assessed on RNA Pico chips in using an Agilent 2100 Bioanalyzer (Agilent, Santa Clara, CA). RNA samples had RNA Integrity Numbers (RIN) ranging from 2.6 to 9.4. One microgram of total RNA was used to construct RNA-Seq libraries using a TruSeq stranded total RNA library prep kit with RiboZero(H∖M∖R) ribosomal RNA reduction (Illumina Inc., San Diego, CA) according to the kit's instructions. RNA fragmentation times were modified based on RNA samples' RIN value to generate inserts of equal size across all libraries.

Twenty RNA-Seq libraries were clustered on an Illumina cBot and sequenced on a HiSeq 1500 platform, in a 2 x 50 base paired end design yielding a minimum of 50 million reads per sample. Five matched pairs of samples (initial biopsy and subsequent surgery) were collected from each of the walnut consuming and control groups.

Reads were trimmed using Trimmomatic v 0.36 [2] to remove low-quality base calls and adapter sequences, and then aligned to the human reference genome GRCh38 using HISAT v2.1.0 [3]. Resulting bam files were sorted and indexed with SamTools v1.3.1 [3], [4], and PCR and optical duplicate reads marked using Picard tools v2.6.0. The numbers of reads mapping to each gene for each sample were counted using the R/Bioconductor package GenomicAlignments, v1.12.2 [5] and the Ensembl gene database for GRCh38, build 84 [6]. Differential gene expression was computed using DESeq2 version 1.10.0 [7], with a statistical model comparing the ratio of expression between surgery and biopsy specimens for the walnut-consuming group to the ratio of expression between surgery and biopsy specimens for the control group, as described in “statistical analyses” below.

### 2.6 Statistical analyses

Differences between groups (walnut or control) in fractions of individual fatty acids as determined by gas chromatography or in clinical parameters were determined by T-test with a significance level of p ≤ 0.05.

It was expected that there would be large interpatient heterogeneity thus the baseline mRNA expression of individual genes would be highly variable between patients. The analyses of biopsy and surgical specimens allowed each patient to serve as her own control. Gene expressions for the sample collected at initial biopsy and the sample collected at surgery were determined and the ratios of these expressions were calculated for each patient. Then the means of the ratio of expressions were compared between the walnut-consuming group and the control group. The comparison was performed using DESeq2, which models the read count per gene using a negative binomial distribution, and moderates the estimated expression changes to account for the dependence on overall read count [7]. Each patient was assigned a unique id within their treatment (walnut or control) group, and the statistical model (equation (1)):

(1) extraction + treatment + patient's group + extraction**:**treatment

was passed to DESeq2, with extraction taking values “biopsy” or “surgery”, and treatment taking values “walnut” or “control”. Genes that were significant for the extraction**:**treatment interaction parameter at a Benjamini-Hochberg (B–H) controlled false discovery rate of 10% were considered to be differentially expressed. The corresponding moderated fold change computed by DESeq2 is an estimate of this parameter, and can thus be considered to be an estimate of the quantities:

(2) gw,sgw,bgc,sgc,b

In Equation (2), ‘g’ represents the expression level of gene ‘g’, the subscripts ‘w’ and ‘c’ represent samples in the walnut and control groups, respectively, and the subscripts ‘s’ and ‘b’ represent samples from surgery and biopsy, respectively. Thus, the ratio of expression of gene ‘g’ from surgical specimens verses biopsy specimens of walnut patients was divided by the ratio of expression of gene ‘g’ from surgical specimens verses biopsy specimens of control patients. These analyses determined whether, across the group, there were significant and consistent changes in the mRNA expression of specific genes due to walnut consumption and provide the input for subsequent Ingenuity Pathway analyses.

### 2.7 Ingenuity Pathway analyses (IPA)

The complex data resulting from RNA seq expression profiling requires complex analyses. Data were analyzed by use of IPA [8]. Final downstream phenotypic effects are due to the balance of positive and negative influences on expression of genes in a pathway. The goal of the IPA Downstream Effects Analysis is to identify genes and the resulting functions that are expected to increase or decrease, given the observed gene expression changes in the experimental dataset [8]. Downstream Effects Analysis is based on expected causal effects between genes and functions; the expected causal effects are derived from the literature compiled in the Ingenuity^®^ Knowledge Base [8]. The analysis examines genes in the dataset that are known to affect functions, compares the genes’ *direction of change* to expectations derived from the literature then issues a prediction for each function based on the direction of changes in the dataset [8]. IPA uses a z-score algorithm to make predictions which is designed to reduce the chance that random data will generate significant predictions [8]. A publication further describing Downstream Effects Analyses can be found at [9].

The p-values for networks were calculated using a Fisher exact test with B–H multiple testing corrections. The networks were generated through the use of IPA [8]. The network score is based on the hypergeometric distribution and is calculated with the right-tailed Fisher's Exact Test with B–H multiple testing corrections. For example, for a network with a p-value of 1 × 10^−30^, the network's score = [-log(Fisher's Exact test result)] = 30. Thus, a score of 30 can be interpreted as meaning that if there were no associations between walnut consumption and the gene expression changes seen in the network, an overlap between the network and the differentially expressed gene set would only occur 1 in 10^30^ times in similar experiments.

Files containing the raw reads, along with a shell script describing the complete data analysis pipeline, were deposited to the Gene Expression Omnibus (GEO) at the National Center for Biotechnology Information (NCBI) and can be obtained via accession number GSE111073. https://www.ncbi.nlm.nih.gov/geo/query/acc.cgi?acc=GSE111073.
