# Supplementary material for: mRNA expression data in breast cancers before and after consumption of walnut by women
Source: Data Brief. 2019 May 23;25:104050. doi: 10.1016/j.dib.2019.104050 (PMC6557731; doi:10.1016/j.dib.2019.104050)
Supplement: Multimedia component 1 [file mmc1.docx]

AUTHORS CONFLICT OF INTEREST DECLARATION

We wish to draw the attention of the Editor to the following facts which may be considered as potential conflicts of interest and to significant financial contributions to this work. Major funding for this research was from the California Walnut Commission (CWC). The CWC had no input on the experimental design, the interpretation of results or the decision to publish the data. All data has been deposited in the Gene Expression Omnibus (GEO) at the National Center for Biotechnology Information (NCBI) and can be obtained via accession number GSE111073. https://www.ncbi.nlm.nih.gov/geo/query/acc.cgi?acc=GSE111073

We confirm that the manuscript has been read and approved by all named authors and that there are no other persons who satisfied the criteria for authorship but are not listed. We further confirm that the order of authors listed in the manuscript has been approved by all of us.

We confirm that we have given due consideration to the protection of intellectual property associated with this work and that there are no impediments to publication, including the timing of publication, with respect to intellectual property. In so doing we confirm that we have followed the regulations of our institutions concerning intellectual property.

We further confirm that any aspect of the work covered in this manuscript that has involved human patients has been conducted with the ethical approval of all relevant bodies and that such approvals are acknowledged within the manuscript.

We understand that the Corresponding Author is the sole contact for the Editorial process (including Editorial Manager and direct communications with the office). He/she is responsible for communicating with the other authors about progress, submissions of revisions and final approval of proofs. We confirm that we have provided a current, correct email address which is accessible by the Corresponding Author.

Signed by corresponding author and for the other authors:

W. Elaine Hardman

Email: hardmanw@marshall.edu
